# Strengthening Biomedical Research and Healthcare in Africa Through Localized Reagent Production

**DOI:** 10.1155/bmri/7361394

**Published:** 2026-03-11

**Authors:** Benedict Ofori, Righteous Kwaku Agoha, Kwamina Nyame, Kwabena Amofa Nketia Sarpong

**Affiliations:** ^1^ Medical College of Georgia, Augusta University, Augusta, Georgia, USA, augusta.edu; ^2^ Department of Biochemistry, Cell and Molecular Biology, College of Basic and Applied Sciences, University of Ghana, Accra, Ghana, ug.edu.gh; ^3^ Department of Biochemistry, Stanford University School of Medicine, Stanford, California, USA, stanford.edu; ^4^ West African Centre for Cell Biology of Infectious Pathogens (WACCBIP), Accra, Ghana

**Keywords:** biomedical research, funding, local manufacturing, reagent production, sustainability

## Abstract

Africa is home to 15% of the global population and bears nearly a quarter of the world′s disease burden. However, it faces significant challenges in biomedical research and healthcare due to limited access to essential reagents. This review examines the critical need for localized reagent production to strengthen biomedical research and healthcare across the continent. The reagent market in Africa is characterized by varying levels of development, and the current production capacity falls short of meeting the biomedical research and healthcare demands. The reliance on imported reagents results in high costs, lengthy procurement processes, and vulnerability to supply chain disruptions. Establishing reagent production facilities across the continent can enhance self‐sufficiency, reduce costs, and improve biomedical research tailored to the unique health challenges faced by African populations. Such efforts can be supported by strategic public–private partnerships and international collaborations, leveraging the successes of the existing pharmaceutical investments across the continent and local capacity‐building initiatives. Regional collaboration, market development, technical training, infrastructure, regulatory frameworks, and sustainable funding are essential to ensure long‐term success. Developing skilled human capital and creating a unified market through initiatives like the African Continental Free Trade Area can attract investment and ensure economic sustainability of local reagent production. Together, these measures can significantly improve biomedical research output and healthcare response capabilities, preparing the continent for future health emergencies.

## 1. Introduction

Africa comprises 15% of the world′s population, bears about a quarter of the global disease burden, yet contributes only 2% of the world′s research output [1, 2]. Projections from the World Health Organization (WHO) and other studies suggest that by 2030, noncommunicable diseases (NCDs) will become the leading cause of death in Africa [3, 4], with prostate and colorectal cancers expected to rise significantly by 2050 [5, 6]. The continent faces challenges with diverse infectious diseases, with the mpox outbreak serving as a notable recent example [7, 8]. Despite these alarming trends, research efforts to tackle these threats remain limited due to challenges such as inadequate funding, infrastructure, and technical expertise [9, 10]. Access to biomedical reagents in Africa is hindered by high costs, complex procurement processes, and lengthy shipping times [11]. These challenges, including international supply chain bottlenecks and excessive shipping costs, have contributed to Africa lagging in global biomedical research output [12–14]. Localized (in‐house) reagents are chemical solutions, biological materials, or other compounds prepared and validated internally within a biomedical research facility or clinical laboratory, or produced by locally based manufacturing companies, rather than purchased as ready‐made products from external suppliers. Africa′s reagent market is expanding, though development varies by country. This growth is driven by local initiatives, partnerships, foreign funding, and the need to improve self‐sufficiency to address global health challenges and strengthen diagnostic capacity in the region [11].

Building capacity to develop localized reagents in Africa and scale up production requires strong collaborations. Research universities in Africa can play a key role by establishing training programs that can equip young scientists with the requisite skills needed to supply the human workforce needed for reagent biomanufacturing [11, 15]. In Latin America, which faces similar challenges with reagent access, initiatives like Reclone have made significant strides [16]. Reclone has supported open science by creating tools like the Research in Diagnostics Deoxyribonucleic Acid (DNA) Toolkit and the Open Enzyme Collection, benefiting 500 researchers in 50 countries. Collaborative efforts are underway to establish a Reclone Hub in Argentina, encourage open biosharing practices, and promote open science in public universities [16]. Additionally, a recombinant protein production center at the University of Buenos Aires is being developed to produce affordable molecular biology enzymes [16]. These examples show that targeted initiatives and collaboration can increase access to essential reagents and build local research capacity. In Africa, similar approaches, tailored to local needs and supported by partnerships, training programs, and investments, could strengthen biomedical research and improve diagnostic capabilities. This review examines the current state of reagent production in Africa, evaluates the barriers to reagent production in Africa, and discusses how strengthening local production could transform biomedical research and enhance the continent′s diagnostic and biomedical research capacities.

## 2. Overview of the Reagent Market in Africa

The reagent market in Africa is underdeveloped and faces many challenges. However, promising efforts are underway to enhance accessibility, promote local manufacturing, and strengthen supply chains to support biomedical research, diagnostics, and industrial applications. Globally, the biochemical reagent market is valued at $37.4 billion in 2025 and projected to reach $81.6 billion by 2035, with North America holding about 40% of the market, followed by Europe and the rapidly growing Asia‐Pacific region with China as a key driver, whereas Africa remains largely left behind [17]. The central goal of creating regional reagent‐manufacturing hubs across Africa is to ensure timely access to essential reagents for diagnostic testing, biomedical research, national laboratories that handle high‐risk pathogens, food and drug regulatory authorities, and emerging biomanufacturing companies. [18, 19]. Collectively, the increasing demand for health and research services on the continent [20] makes these sectors viable consumers of reagents, creating a sustainable market that will be economically attractive for bringing investors into local manufacturing. Although this list is not exhaustive, a careful assessment of the diverse sectors that rely on reagents and the scale of their demand will be essential for establishing a self‐sustaining regional manufacturing ecosystem capable of strengthening the continent′s readiness for current and future health challenges.

International organizations have funded most African reagent production hubs, as local financial support is often lacking [21, 22]. Some argue that the region does not prioritize supporting such initiatives, despite the clear benefits of scaling up reagent production [23]. In developed countries, the majority of reagent‐producing companies are not state‐owned [24], yet they operate successfully because robust demand, supportive government policies, including intellectual property protections, tax reductions, and other incentives, and mature supply chains create a conducive environment for private investment. A clear example is seen in the United States, where the Internal Revenue Service allows firms to claim 20% of their R&D expenses as a tax credit [25]. Also, in Europe, a new Biotech Act is aimed at closing the innovation gap by making it easier to move discoveries into production, a change that is expected to boost the reagent industry through clearer regulations, faster approvals, and increased investment in biomanufacturing [26]. Although documented efforts in Africa remain limited, available evidence suggests that some government‐led partnerships with international financiers and development organizations are beginning to support local biomanufacturing initiatives. In 2020, a collaboration between the European Investment Bank and the Foundation for Innovative New Diagnostics in Mauritius allocated €47.5 million to establish a plant‐based protein manufacturing facility [21]. This plant is aimed at producing reagents needed to produce 100 million vaccines per month for distribution across Africa. Beyond supplying crucial reagents for testing and treating diseases like severe acute respiratory syndrome Coronavirus 2 (SARS‐CoV‐2), human immunodeficiency virus (HIV), Ebola, dengue, measles, and Yellow fever, the project also created skilled jobs in a high‐unemployment region. This example shows that while private investors will play a major role in Africa′s reagent manufacturing market, supportive and predictable government policies are important to make these ventures viable and to ensure a market that will see their long‐term growth and sustainability. Building capacity for reagent production can significantly benefit countries across Africa by addressing regional needs at lower costs, promoting sustainability, and improving biomedical research and healthcare [9, 11].

According to WHO Africa′s AFR/RC73/7 document, which outlines the African regional strategy for diagnostic and laboratory services from 2023 to 2032, only 30% of African health facilities have the equipment and reagents needed to perform basic diagnostic tests [27]. This is a big issue because most countries in Africa are low‐ and middle‐income countries (LMICs) and already face a high burden of infectious diseases and NCDs [28, 29]. The lack of capacity to develop reagent production facilities in the region affects research progress, which became evident during the COVID‐19 pandemic. Many cases were never tested, so the true extent of the virus′s spread in the region remains unknown [30]. Even though funding was available during the COVID‐19 pandemic due to several donations, European countries banned the purchase of reagents by African researchers and health facilities that needed these crucial reagents for testing, identifying circulating strains, and surveillance purposes [31]. This demonstrates how critical it is for African countries to build local capacity and create an enabling environment that attracts both local and foreign investors engaged in R&D to set up reagent production facilities, ensuring the region has timely and reliable access to these essential materials. Apart from the reduction in the delay and cost involved in purchasing reagents from the international market, the region would also be positioned to be ready for future disease outbreaks and pandemics [31, 32].

There are a few notable companies across Africa, such as Cape Bio Pharms, Inqaba Biotechnical Industries Pty Ltd, Merck Life Science Africa, BIOCOM Africa, AzarGen Biotechnologies in South Africa, and Sysmex West and Central Africa Ltd, that develop reagents locally. In North Africa, few companies including Misr Technology for Biological Industries and Ultracare Diagnostics in Egypt specialize in producing high‐quality IVD reagents rather than merely distributing products from international firms. In countries where these firms operate, they often cannot meet local demand, forcing many to rely on external suppliers [11, 33, 34]. This highlights the growing need for facilities to produce essential reagents for both research and diagnostics within the continent. Recently, Wondfo Biotech, a leading company in point‐of‐care (POC) diagnostics, signed a memorandum of understanding with Nigeria′s Presidential Initiative for Unlocking the Healthcare Value Chain (PVAC) to localize diagnostic reagent production [35]. In Egypt, a significant market gap exists, with reagent imports totaling 131 million USD compared with only 1.51 million USD exports, resulting in a trade deficit of 129.49 million USD [34]. To address this, Egypt has launched a feasibility study to produce reagents locally for diagnostic purposes. Furthermore, researchers in various institutions and clinical laboratories have made efforts to develop customized in‐house assays to meet specific needs; however, these initiatives often remain confined to individual institutions and are not widely adopted.

Data from the World Integrated Trade Solution show that countries like Togo, São Tomé and Príncipe, Ghana, Cape Verde, Djibouti, Seychelles, Central African Republic, and Angola were among the top importers of laboratory reagents in 2023. The biotech firms and reagent manufacturing hubs in African countries primarily act as subsidiaries of their parent companies outside the continent or as distributors for large international companies or partners. Also, the materials required for local production are often heavily taxed through value‐added taxes (VATs) and import duties [14], creating additional challenges for these firms. This model does little to support cost‐effective, regionally tailored reagent production. These challenges could be addressed through creating an enabling environment to attract key private investors to set up these facilities. For instance, enzymes, which make up 70%–90% of the cost of many diagnostic assays [36], are critical but often not produced locally. There have been minimal efforts to clone and express these enzymes within the region. One example is the loop‐mediated isothermal amplification (LAMP) assay, used for diagnosing diseases like malaria and schistosomiasis, which relies on the costly *Bacillus stearothermophilus* (BST) enzyme. A study in Ghana demonstrated that local production of reagents for LAMP assays could reduce the cost of manufacturing BST enzymes to just 5% of their current price in LMICs [37] showcasing the potential for cost savings and increased self‐sufficiency in reagent production.

Local reagent production is also important for addressing the genetic diversity and polymorphism unique to African populations, especially for genomic tests and assays for pathogen detection and antibody recognition [38–41]. Although several biotech firms and labs that focus on producing diagnostic services in Africa focus on assays for genetic testing, none of them manufacture genomic test kits, and most genetic testing facilities rely on imported commercial kits [42]. Commercial diagnostic reagents produced outside Africa may fail to accurately identify diseases due to factors like unique climate conditions, genetic profiles, and varying disease prevalence, as African regional validation studies are often lacking during their development [40, 43, 44]. Locally produced reagents tailored to the specific needs of the population can improve reliability and enhance diagnostic accuracy. In Burkina Faso, researchers have developed a red blood cell (RBC) reagent for antibody detection to mitigate the high costs and short shelf life of imported reagents [45]. This locally developed RBC reagent demonstrated a concordance rate of 97.5% with imported RBC panels and had a kappa agreement coefficient of 0.815. Additionally, it offered a 4‐week shelf life, addressing delays associated with importing reagents. The reagent shows the potential to reduce the risk of RBC incompatibilities in blood transfusions in landlocked countries. Table 1 presents studies that have utilized in‐house reagents. However, we excluded many other studies as they primarily focused on optimizing reaction conditions rather than producing reagents locally. This highlights the limited efforts dedicated to reagent production on the continent, even at the level of in‐house use within research and clinical laboratories.

**Table 1:** Summary of selected in‐house reagents developed in Africa and their comparative performance.

Country	Reagent developed	Diagnostic/research purpose	Performance compared with commercial reagents	Unique value	Reference
Burkina Faso	RBC panel	Antibody detection for blood transfusions	The concordance rate with the commercial RBC panel was 97.5%, with a kappa agreement coefficient of 0.815	Locally tailored reagents with shorter production and supply time	[45]
Botswana	Integrase drug resistance assay (IH‐Int)	Detection of integrase strand transfer inhibitor (INSTI) resistance mutations in HIV‐1C for treatment monitoring	Amplified 100% of plasma samples with viral loads > 1000 copies/mL, compared with 85.4% success rate with the ViroSeq HIV‐1 integrase assay: 99.8% sequence identity	Cost‐effective (25% of commercial assay cost), tailored to HIV‐1C, higher success rate due to HIV‐1C‐specific primers, suitable for resource‐limited settings	[46]
The Gambia and Senegal	SYBR green‐based qRT‐PCR assay for detecting HBV genotypes	Detection of hepatitis B virus (HBV) genotypes in sub‐Saharan African population	High accuracy with viral loads ranging from 2 to 8 log copies/mL; compared with Abbott RealTime HBV/Abbott m2000sp/rt test and COBAS TaqMan HBV Version 2.0	Highly cost‐effective (two to three times cheaper than commercial kits), covers HBV genotypes A‐F, sensitivity down to 30 copies/mL, efficient for large‐scale epidemiological studies, similar run time as commercial assays	[47]
Ethiopia	FITC/anti‐FITC GACELISA (glycosylated antigen capture enzyme‐linked immunosorbent assay)	Measurement of measles‐specific IgG in oral fluid	High sensitivity (97.4%) and specificity (90%) compared with commercial serum Behring ELISA kit.Strong concordance with serum ELISA (*κ* = 0.837).	Noninvasive oral fluid sample compared to the commercial serum Behring ELISA kit.Stable antigen under varied conditions (up to 30 days). Cost‐effective and field‐suitable alternative.	[48]
Egypt	In‐house ELISA and LFA	Detection of *Mycoplasma gallisepticum* (MG) infection in poultry	The in‐house ELISA demonstrated a strong correlation with commercial ELISA kits. The LFA exhibited a sensitivity of 77.5% and a specificity of 92% when compared with PCR.	This represents the first development of an LFA for diagnosing MG in chickens in Egypt, providing a rapid diagnostic tool for detecting MG infections.	[49]
Egypt	DNA extraction kit	Diagnosis of active human brucellosis	DNA extracted from in‐house extraction kits showed higher sensitivity, specificity, and accuracy after PCR compared with commercial extraction kits.	More economical alternative to commercial kits, with high sensitivity, specificity, and accuracy. PCR showed 100% positivity in patients with positive blood cultures.	[50]

## 3. Strategies for Localizing Reagent Production in Africa

The focus of biotechnology in Africa has largely centered on agriculture and food systems, driven by challenges like climate change, pests, and diseases. However, there is limited data on efforts to harness Africa′s vast microbial resources, agricultural byproducts, marine and mineral resources, animal‐derived materials, local plants, and herbs, as well as the rich human and animal microbiomes and other accessible raw materials to develop essential reagents for research and healthcare. The biotechnology industry in Africa holds the key to transforming the health of its people and advancing scientific research. Reagents used in biomedical research vary widely, but here, we highlight key methods used to produce essential reagents to support research on infectious and non‐infectious diseases, as the region continues to fall behind global efforts.

### 3.1. Recombinant Protein Expression

Recombinant protein expression has been utilized in numerous scientific research and biotechnological applications. Among its diverse uses, it is predominantly employed in the production of vaccines, monoclonal antibodies (mAbs), hormones, and glycosylated proteins [51]. This biotechnological technique facilitates the large‐scale production of biologically active proteins from DNA templates in an expression system [52]. Genes of interest are identified, isolated, and characterized, then inserted into vectors using recombinant DNA technology. These vectors are introduced into host cells, which use their replication machinery to produce the desired proteins. Several expression systems are available for the production of recombinant proteins; in the following section, we focus on bacterial and yeast systems, as well as mammalian cell lines.

#### 3.1.1. Bacterial Expression Systems

In contrast to many chemically synthesized pharmaceuticals, proteins and peptides that serve as reagents for many biomedical research applications are primarily made in bacterial host cells. However, cell‐free expression systems are available. Bacterial expression systems are particularly suitable for nonglycosylated or non–posttranslationally modified proteins [53]. Recent evidence notes otherwise. Bacterial expression systems, particularly *Escherichia coli* (*E. coli*), have shown promise in producing glycosylated proteins by transferring the N‐linked glycosylation system from *Campylobacter jejuni*. This system enables *E. coli* to glycosylate proteins such as AcrA with native and nonnative glycans, facilitating the production of glycoproteins for diverse applications [54]. Bacterial expression systems present an efficient and cost‐effective solution for generating proteins [55]. These expression systems have produced several essential reagents for infectious disease research. Recombinant proteins such as antigens and enzymes are among the most common products of these systems [56]. For example, malaria research in Africa could benefit from the production of recombinant *Plasmodium* antigens for diagnostic tools and vaccine development [57]. In addition to antigens and antibodies, bacterial expression systems are effective for producing bacterial toxins used in vaccine development [58]. Furthermore, enzymes such as polymerases and restriction endonucleases used as reagents in molecular biology applications can be produced locally using *E. coli*. These enzymes produced locally will reduce the reliance on expensive imports, thus supporting a range of diagnostic and research activities. Bacterial expression systems offer several advantages, including simplicity, cost‐effectiveness, scalability, well‐characterized and engineerable properties, and regulatory acceptance [59]. Bacteria such as *E. coli* are biologically and physiologically less complex than eukaryotic systems, rendering them a cost‐effective choice for recombinant protein production [60]. Furthermore, they are well‐studied organisms with fully sequenced genomes, enabling genetic engineering to generate novel functionalities [61]. Other commonly used bacteria apart from E. coli are *Lactococcus lactis* and *Pseudomonas* species (*P. aeruginosa*, *P. putida*, and *P. fluorescens*), and these species have been isolated and characterized in several countries across the continent [59]. Adopting bacterial expression systems for local protein production can transform research efforts and vaccine development.

#### 3.1.2. Mammalian Cell Lines

Mammalian cell lines are widely used to produce recombinant proteins and mAbs for biopharmaceutical applications, including therapeutics, vaccines, and diagnostic reagents. Chinese hamster ovary (CHO) cells, capable of generating nonimmunogenic antibodies and exhibiting glycosylation patterns very similar to those of human antibodies, currently account for about 70% of global monoclonal antibody production and ribonucleic acid–binding proteins (RBPs) [62]. In Africa, there is growing evidence of CHO cells being successfully used in various downstream applications, which demonstrates their potential in the local production of mAbs and RBPs. Scaling up the use of CHO cells in Africa could improve the manufacturing of mAbs, thereby supporting a broad range of biopharmaceutical products and protein expression‐related research, enhancing the region′s capacity to meet research and healthcare needs. These cell lines capture the genetic and epigenetic traits of local populations and offer an accurate model for studying diseases such as malaria, sickle cell disease, and cancers [63]. Local production of these proteins could reduce reliance on imported reagents. Although many proteins are similar across humans, small genetic differences can lead to variations in protein structure, expression, and modifications [64]. Also, locally derived cell lines could produce proteins that are tailored to the unique characteristics of African populations, thereby improving diagnostic services and supporting therapeutic development. Although producing these antibodies and proteins requires expertise in molecular biology and strict regulatory adherence, establishing proper regulatory structure and enhancing development and standardization would enable mass local recombinant protein production that transforms biomedical research and healthcare on the continent.

#### 3.1.3. Yeast Expression Systems

Yeast expression systems, particularly *Saccharomyces cerevisiae* and *Pichia pastoris*, are widely used in research and industry due to their versatility and ability to perform posttranslational modifications such as glycosylation [65]. This capability is essential for producing biologically active glycoproteins, including antibodies and cytokines that require proper folding and glycosylation to function effectively [66]. As a result, yeast systems are commonly employed in the production of therapeutic proteins and industrial enzymes [67]. In resource‐limited settings, yeast expression systems offer significant adaptability. They are relatively inexpensive to maintain compared with mammalian cell cultures, requiring simpler growth media and infrastructure [68]. Yeast can grow rapidly and to high cell densities in basic fermentation setups, suitable for large‐scale protein production [69]. Additionally, their tolerance to varying environmental conditions reduces the need for highly controlled laboratory environments. Furthermore, yeast systems are highly adaptable to genetic manipulation, allowing researchers to optimize protein yields and tailor strains to specific needs [70]. The simplicity of yeast culturing, combined with the availability of well‐characterized vectors and protocols, makes it an accessible option for laboratories in developing regions like Africa.

### 3.2. Assay and Kit Development

Locally developed assays and test kits are essential for improving health systems, as they provide fast and accurate diagnoses that help control the spread of diseases. This is especially necessary in regions where vaccines are not available for many devastating diseases. There is a pressing need to produce diagnostic reagents locally in Africa to support the development of test kits, including POC devices, lateral flow assays, and molecular diagnostics. Such efforts would significantly enhance biomedical research and strengthen healthcare across the continent.

#### 3.2.1. POC Devices

POC devices provide quick and accessible testing for electrolytes, enzymes, drugs, and biomarkers for infectious diseases, making them valuable tools for healthcare [71, 72]. In Africa, the reliance on imported POC testing kits has created significant challenges due to their high costs and limited affordability for those in need [73, 74]. Although POC tests can potentially improve diagnostic access, their effectiveness is often undermined by cost barriers and limited availability in low‐income settings. Moreover, studies have shown that the performance of these POC tests can be suboptimal in African contexts, as regional disease prevalence, temperature, and humidity can significantly affect their performance [75, 76]. A survey across five African countries revealed a high incidence of false positive results in rapid diagnostic test (RDT) kits used in detecting HIV, indicating a need for local validation tailored to specific populations and disease subtypes [77]. This unreliability can discourage individuals from seeking testing or treatment, further hindering efforts toward addressing the pressing public health challenges in the region. The test components of these POC test kits are mostly reagents, ranging from buffers, enzymes, antibodies, and antigens to substrates. These components are essential for ensuring the tests work reliably and accurately. However, because many of these reagents are imported, they significantly add to the overall cost of the test kits and can cause delays in supply. Producing these reagents locally could reduce costs, streamline the supply chain, and develop tests that are better tailored to the specific health challenges and environmental conditions found in Africa. This approach would make diagnostics more affordable and accessible to promote local capacity building and innovation in biomedical research.

#### 3.2.2. Lateral Flow Assays

Lateral flow assays are simple and quick tests designed to detect specific substances in a liquid sample, like proteins or biomarkers of a wide range of pathogens [78]. The underlying principle involves the migration of the test sample along a strip containing immobilized test components, such as antibodies or antigens, which specifically bind to the target substance. A visible line or marker forms on the strip if the target substance is present, indicating a positive result. These tests are widely used because they are easy to use, do not require complex equipment, and give results in minutes. In Africa, RDTs for various tests, including pregnancy and kits for infectious diseases such as malaria, SARS‐CoV‐2, and HIV use lateral flow technology. The lateral flow assay market is projected to grow at a compound annual growth rate of 8.29% by 2029 [79]. However, despite Africa′s population being expected to double by 2050, with one in four people globally anticipated to be Africans [80], the market for lateral flow tests in the region remains minimal to nonexistent. Furthermore, this market′s Top 12 major players are all based outside Africa, limiting local accessibility and affordability [79]. Proper planning and commitment from stakeholders and researchers across the continent could lead to the development of easy‐to‐use kits. Regulatory bodies must ensure that the antibodies used are highly specific, sensitive, stable, and pure to maximize effectiveness.

#### 3.2.3. Molecular Diagnostics

Molecular‐based assays are regarded as gold standards for detecting high‐priority diseases due to their superior sensitivity and specificity [81, 82]. These assays analyze molecules such as DNA, ribonucleic acid (RNA), or proteins in tissue and fluid samples to diagnose conditions like infectious diseases and cancer [83]. Despite their efficiency, molecular diagnostics are not widely accessible in Africa [42, 84]. They are typically constrained to research settings or used for tests of national significance because of their high cost. Most of these assays depend on imported components, particularly reagents, which significantly raise prices and limit accessibility [85]. However, many of these components could be produced locally in Africa, decreasing costs and enhancing availability. The COVID‐19 pandemic underscored the gap in molecular diagnostics, as many individuals in Africa remained untested due to a lack of affordable testing options [84]. Similarly, in 2013, a study in South Africa indicated that laboratory costs for the Xpert MTB/RIF test, a molecular diagnostic tool for tuberculosis, were $14.93 per sample, whereas the MTBDR plus line probe assay cost $23.46 per sample [86]. These costs were higher compared with $16.88 per sample for conventional liquid culture‐based methods. The primary driving factor of these costs, accounting for 60%–80%, was the price of consumables, including test reagents. The Xpert MTB/RIF test is recognized for its high sensitivity and specificity, making it invaluable in regions with high TB prevalence [86, 87]. Although Xpert MTB/RIF comes with a cartridge that houses the reagents, ensuring its continued use in resource‐limited settings would necessitate local production of some consumables. This would require collaboration with the developers to maintain the quality and integrity of the diagnostics while making them more affordable and accessible. In Ghana, researchers have successfully expressed and purified BST, the polymerase enzyme used in LAMP, which was previously only available commercially [37]. This was achieved using nickel His‐Bind resin. However, none of these locally produced proteins have been commercialized for use in national or regional laboratories, which remains a significant concern. Efforts to produce reagents for molecular diagnostics can enhance surveillance of high‐priority diseases and those that require highly sensitive and specific assays. This will better prepare the continent to address these diseases and enhance biomedical research.

### 3.3. Biochemical Reagent Synthesis

Biochemical reagents are essential for driving chemical reactions that occur in living organisms. These reagents include enzymes, peptides, stains, buffers, media, histology products, and more. The widespread use of enzymes in many research labs highlights the need for local production. In addition, almost every biomedical research lab relies on buffers and media for experiments and cell culture.

#### 3.3.1. Enzyme Production

Enzymes are widely used in drug synthesis, cosmetics, disease diagnostics, and various chemical reactions [88, 89]. They are grouped into classes based on their properties and functions, with hydrolases, commonly employed in techniques such as PCR, cloning, protein expression, RNA sequencing, and enzyme‐linked immunosorbent assays, making up over 75% of all enzymes [90]. Leading biotech companies like BASF (Germany), Novonesis, formerly Novozymes (Denmark), DuPont (United States), DSM (The Netherlands), Advanced Enzyme Technologies (India), and Roche (Switzerland), dominating the global enzyme production market, are all based outside Africa [91, 92]. Africa has abundant resources for enzyme production, as most enzymes are derived from fungi (60%), bacteria (24%), yeast (4%), and plants or animals (10%) [92]. However, there are limited local efforts to produce enzymes commercially. This is largely due to a lack of enzyme engineering initiatives, which are responsible for about 90% of all commercial enzymes through techniques like metagenomics, proteomics, and genome mining [92, 93]. Natural enzymes often fail under harsh conditions [94], making engineering strategies such as directed evolution, site‐directed mutagenesis, truncation, and terminal fusion essential [95]. Despite Africa′s rich microbial diversity [96], the continent relies heavily on imported enzymes for biomedical research. However, recent initiatives are making progress in enzyme production locally. In 2013, the Enzyme Manufacturing Masterclass brought researchers and bio‐entrepreneurs from Kenya and Ethiopia to learn skills and open‐source protocols for local enzyme production. The aim was to reduce reliance on imported reagents and equipment while promoting innovation and self‐sufficiency, and participants were able to discover how to produce polymerases for PCR, a powerful molecular biology technique [97]. Also, Afrizymes in South Africa manufactures enzymes from local microorganisms for industries like wastewater treatment, textiles, and food processing [98]. Their efforts reduce dependency on imports, lower costs, and support environmental sustainability, which has significant implications for reducing the spread of infectious pathogens, as these untreated wastewaters are known to harbor pathogens [99]. Furthermore, in South Africa, there have been efforts to produce cellulase enzymes using locally isolated *Trichoderma* and *Aspergillus* species grown on banana pseudostems through solid‐state fermentation, highlighting the potential of using agricultural waste for sustainable enzyme production [100].

#### 3.3.2. Buffer and Media Preparation

Buffers are primarily composed of water and salts and are essential in biomedical research and healthcare. They mimic biological solutions and are used in various biochemical and biophysical studies [101]. One of the most commonly used buffers is phosphate‐buffered saline (PBS), made from disodium phosphate (Na_2_HPO_4_^−^) and potassium dihydrogen phosphate (KH_2_PO_4_^−^), with a pH of 7.4 [101, 102]. Despite being made from readily available salts, laboratories often rely on commercial, preformulated PBS solutions or powder due to trust in their nuclease‐free quality, which is important for biochemical and molecular assays [103]. The current cost of commercial PBS, such as a 500‐mL solution priced at $83.10 on Thermo Fisher′s website, is significant for many laboratories across the continent. However, PBS can be easily prepared in‐house by following specific protocols to ensure it is nuclease‐free. In addition to buffers, media are essential for supporting the growth of cells, bacteria, fungi, and cell cultures, particularly in infectious disease and cancer research [104, 105]. These nutrient‐rich substances contain peptones, extracts, sugars, growth factors, minerals, and vitamins, which are often available locally. However, a narrative review revealed that laboratories in LMICs face challenges in media preparation, including a lack of clean water, unstable power supply, high environmental temperatures, dust, inexperienced staff, and poor‐quality consumables [106]. The review also suggested potential solutions, such as centralized local production, use of alternative blood sources like anticoagulated hair sheep blood, enhanced roles for national reference laboratories and clinical microbiologists, promotion of the WHO Essential Diagnostics List, support for professional societies, and stronger regulatory frameworks.

With funding from the European and Developing Countries Clinical Trials Partnership (EDCTP) 2 Simplified Blood Culture System (SIMBLE) project, a culture media production facility was established in Spain and later installed in Benin, significantly reducing the costs associated with shipping reagents from outside the region [107]. Such initiatives are essential for enhancing local research capacity and accessibility to vital resources. Additionally, Stellenbosch University, through a self‐initiated $66 million fund, has opened the Biomedical Research Institute (BMRI), hailed as Africa′s most advanced biomedical research center [108]. Scientists at BMRI attribute its success to effective collaboration and engaging the right stakeholders. They emphasize that, with nearly half of Africa′s population under 25 years old, building local research capacity has the potential to achieve remarkable progress and foster future Nobel laureates from the continent.

## 4. Challenges in Local Reagent Production in Africa

The production of reagents in Africa is a transformative approach that could pave the way for stronger health systems on the continent. However, these efforts cannot succeed without establishing robust infrastructure, regulatory frameworks, and capacity‐building initiatives. Importantly, local reagent production should be understood within the broader political economy of the existing pharmaceutical and biomedical manufacturing across the continent, where private firms and not governments are the primary investors and operators [109]. The region faces several challenges that might hinder large‐scale reagent production, but addressing them will create an environment conducive to local manufacturing and innovation and ultimately boost biomedical research capacity in the region.

### 4.1. Infrastructure and Equipment

One of the major challenges that countries in Africa face is the infrastructural deficit needed for biomedical research [9]. With a high disease burden, low gross domestic product (GDP) per capita, and limited government funding, access to basic laboratory equipment remains a major hurdle [23, 110]. A survey of 130 life sciences researchers across 20 African countries revealed that prices for molecular biology reagents and equipment, such as DNA extraction kits and SARS‐CoV‐2 diagnostic assays, were 25%–116% higher than manufacturer list prices in the United Kingdom [111]. This forces many researchers to rely on substandard protocols and outdated methods [112], which compromises the quality of their studies.

The lack of infrastructure is a widespread issue across the continent and must clearly be distinguished from the requirements for commercial reagent manufacturing, which demand a higher level of capital investment and technical expertise for use of complex equipment [113]. Although many African laboratories struggle to access basic equipment, commercial‐scale production requires purpose‐built facilities operated by specialized reagent manufacturers, rather than governments or academic laboratories. Aside from the benefits that large‐scale manufacturing through private partnerships would bring, laboratories that need to produce small batches of essential reagents for in‐house use will still face major hurdles. This is due to the lack of modern facilities with clean rooms for sterile reagent production, raw material storage, and handling. This distinction highlights two parallel challenges that lie in improving laboratory‐level capacity for research use and creating industrial conditions attractive to private manufacturers capable of producing and supplying reagents on a large scale.

Also, most laboratories often lack the infrastructure for effective quality control checks [114]. This can affect testing and validation studies on reagents that would be produced internally to meet international standards. Furthermore, most laboratories on the continent lack proper waste management systems, a challenge exacerbated by the regional burden of waste management [115]. As a result, they often rely on expensive external services or store chemical waste for extended periods, increasing both costs and contamination risks. Despite the growing global demand for nuclease‐free water in scientific research, the region′s lack of high‐quality water purification systems hinders its production from local water sources [116]. As a result, most laboratories depend on imported nuclease‐free water, which is costly and could pose a challenge for reagent production on a commercial scale, as the preparation of these reagents requires high‐purity water. Additionally, cold storage facilities for temperature‐sensitive reagents are scarce in the region, which already struggles with adequate storage for perishable foods and temperature‐sensitive pharmaceuticals [117]. This has contributed to approximately 1.5 million preventable deaths annually [117, 118], underscoring the significant challenges researchers and future manufacturers would face in storing and distributing reagents after production.

Addressing these infrastructure gaps requires targeted government action, not to directly establish reagent manufacturing factories, but to create an enabling environment that attracts industrial investors capable of commercial‐scale manufacturing. The local pharmaceutical manufacturing industry, which has existed since the 1970s and has long been the focus of calls to strengthen local production due to its limited capacity to supply even one‐third of the medicines needed on the continent, shows that successful localization depends on policies that reduce risk for private investors, such as clear regulations, reliable markets, and supportive government incentives [119, 120]. An enabling environment would involve governments prioritizing investments in research infrastructure, including modern laboratories, clean rooms, quality control facilities, waste management systems, and cold storage. Reducing import duties and taxes on essential equipment would make them more affordable [14]. Also, access to stable electricity, which is a major problem on the continent [121] must be addressed as these facilities would require constant and stable supply of power for their daily production activities. Researchers already face complex procurement processes and lack direct access to equipment manufacturers, relying on intermediaries without opportunities to negotiate better terms [14, 122]. Governments through their regulatory bodies and in partnership with research institutions, universities, and healthcare authorities must simplify procurement procedures to ensure researchers and future reagent manufacturers can easily purchase the tools they need once they set up. Building this infrastructure is critical for scaling up local reagent production, strengthening Africa′s biomedical research capacity, and achieving self‐sufficiency in the region. Governments, stakeholders, and international funders must work together to support these efforts and improve healthcare outcomes across the continent.

### 4.2. Financial and Human Resources

Global health crises continue to pose significant challenges, yet limited attention is given to the personnel at the forefront of addressing these issues [123]. The 2023 WHO Global Observatory on Health highlighted significant disparities in research capacity between high‐ and low‐income countries [124]. The report showed that high‐income countries have 56 times more health researchers per million inhabitants than low‐income countries and attributed this imbalance to the lack of educational institutions dedicated to training research scientists in low‐income regions when compared with the stronger education systems, sustained funding, and closer links between academia and industry in high‐income countries. Furthermore, low‐income countries receive only a small fraction of global research funding. For example, just 0.2% of all grant funding for NCDs and 0.6% for neglected tropical diseases is allocated to institutions in these regions. One of the most important partnerships driving innovation is the industry‐academia partnership, where companies rely on well‐trained researchers from universities while also providing funding, infrastructure, and practical experience that strengthen academic research [125]. In Africa, persistent funding gaps caused by limited government support for biomedical research in universities will reduce the ability of research institutions to serve as reliable partners and talent pipelines for future private reagent biomanufacturing companies. Most governments allocate less than 1% of their annual budget to research [126], which is concerning, especially with international donors pulling back funding. No country has yet met the 1% target. Even South Africa, the continent′s biggest investor in research and innovation, has only reached a peak of 0.85% of its GDP.

Efforts to address these challenges include initiatives like the African Centers of Excellence, a collaboration between the World Bank and African governments aimed at establishing research centers to transform higher education and train more scientists [127]. Although these efforts are commendable, challenges remain. Many young researchers in Africa report a lack of mentorship, limited early‐career funding, and inadequate institutional support [128]. A recent survey of 1000 young African researchers found that 80% intended to leave the continent [129], reflecting a significant “brain drain”. This shortage of skilled personnel could severely impact efforts to produce localized reagents for biomedical research, as trained experts are essential to ensuring high‐quality products that meet international standards. To address this, African governments and institutions must prioritize investments in higher education and expand opportunities for advanced degrees. Specialized training programs in collaboration with biomanufacturing companies and research institutions should also be developed to equip researchers with the skills needed for large‐scale reagent production. In addition, financial incentives such as competitive salaries and benefits are critical to retaining skilled researchers and, hence, must be prioritized. Improving working conditions and creating attractive environments in research institutions will make them more appealing compared with opportunities abroad. These combined efforts can help build a strong workforce to drive localized reagent production, enhance research capacity, and contribute to addressing health challenges in the region.

### 4.3. Regulatory and Ethical Considerations

A stable supply of high‐quality reagents in Africa would depend on strong regulatory frameworks and ethical practices. Currently, there is no dedicated agency specifically regulating reagent production. Although regulatory bodies like the African Medicine Agency set up by the African Union and the recent Africa Centre for Disease Control (CDC) partnership with the United States Pharmacopeia (USP) Convention help oversee pharmaceutical products [130], the absence of clear and harmonized regulatory pathways for reagents increases uncertainty for private manufacturers considering investment in the region.

To ensure quality, the raw materials must be of high grade, meet international standards, and be fully traceable, especially those from plants and animals. A robust system for risk assessment and quality control should be established to monitor production and approve reagents for commercial use. From a political economy perspective, the predictability of the regulatory bodies across the various countries in the region will be a key determinant of whether multinational and regional firms choose to locate production facilities in African countries [131]. Additionally, the regulatory network must guarantee a reliable supply chain so that these reagents reach the intended users. From an ethical standpoint, reagents derived from animal or human sources need to receive proper ethics board approval. Also, local producing plants could participate in international proficiency testing to ensure that the reagents produced are of high standards and follow international regulatory standards for the production of specific reagents. Strengthening these regulatory and ethical systems would reduce investment risk and enhance confidence among both local and foreign reagent manufacturers.

## 5. Strategies and Recommendations for Strengthening Reagent Production in Africa

Although this may be new to the continent, it is essential to strengthen biomedical research, which is key in guiding clinicians on disease trends and treatment options. However, successful reagent production requires well‐planned models and strategies. We propose the following key recommendations and strategies that may support and enhance African reagent production efforts.

### 5.1. Building Local Capacity

Currently, many African countries lack the industrial ecosystem and enabling conditions needed to attract large‐scale biomanufacturing due to the absence of industrial policies that can bring investors capable of setting up mass reagent production facilities [132]. Building the capacity needed to achieve the mass production of reagents across the continent will strengthen biomedical research, help close the gap in biomedical research with Western countries, and ensure that the continent is prepared for unexpected challenges like pandemics. As highlighted in the World Bank report *Microbes and Money*, researchers within a country are best positioned to address health emergencies if they are provided with the right support and resources [133]. Effectively preparing for future pandemics and mitigating the risks posed by outbreaks like Ebola and COVID‐19 will depend significantly on the availability of accessible reagents for testing populations and quickly identifying those at risk to enable prompt responses. Through collaboration with local and international partners, Tanzania recently launched its first localized reagent production facility capable of producing 10,000 barrels of high‐quality in vitro diagnostic reagents [134].

Building this capacity will also depend on strengthening human capital, and several programs already exist that can be leveraged for this purpose. For example, the Mastercard Foundation′s $45 million partnership with The Institut Pasteur de Dakar in Senegal, the African STARS Fellowship Programme funded by Mastercard Foundation, and the recent GIZ‐CSIR collaboration in South Africa are training young Africans in biomanufacturing especially to boost the continent′s effort to produce vaccines locally [135, 136]. In Ghana, similar efforts include the recent establishment of the National Vaccine Institute and the collaboration between the West African Centre for Cell Biology of Infectious Diseases at the University of Ghana and GIZ, which aim to advance biologics and vaccine production [137]. These initiatives can be expanded and tailored to support reagent manufacturing, providing the skilled workforce needed by both local laboratories and incoming private manufacturers.

Stakeholders must commit to building manufacturing capacity, from small‐scale output in research and health laboratories to attracting large reagent manufacturing companies that can supply laboratories unable to produce reagents in‐house for small‐scale purposes. Training programs should be supported through collaborations with experienced institutions to transfer technology and specialized knowledge, fostering innovation suited to local needs [138, 139]. Creating an enabling environment for reagent manufacturers would include upgrading existing facilities and setting up new, modern ones where required [140]. Furthermore, ensuring the proper maintenance of production equipment is necessary to minimize disruptions and ensure a steady supply of reagents [141, 142]. In instances where obtaining equipment and capacity for localized reagent production is challenging, alternative approaches can provide innovative and practical solutions. For example, bacterial incubators and inexpensive chemical desiccants have been developed to dry protein‐expressing bacteria, eliminating the need for expensive and complex lyophilizers [143]. This approach produces stable reagents comparable to commercial enzymes, capable of remaining viable at ambient temperatures for months, and is successfully implemented in diverse local settings, including Ghana and Cameroon.

Lastly, market viability is a key factor in attracting private investors [144]. The successful setup of reagent production facilities across the continent will depend on countries demonstrating a clear and sustainable market. The African Continental Free Trade Area initiative, which has already been promoted to reduce medicine costs through pooled procurement and local production, also provides an opportunity to create a unified continental market [20, 145]. This can enhance production efficiency, allow manufacturers to supply multiple countries efficiently, and ensure that local reagent production is economically sustainable, meeting the growing demand from research institutions, laboratories, and healthcare facilities across Africa.

### 5.2. Technology Transfer

Technology transfer has played an important role in advancing biomedical research, though it comes with challenges. It is a structured process that ensures the smooth transfer of procedures, documentation, and technical expertise from development to production or between manufacturing sites. This process can help simplify complex equipment and techniques, making them more suitable for the specific needs of a region. In various African countries, researchers and future biomanufacturing companies could take the lead in integrating technology transfer into their workflows. They must establish genuine partnerships that benefit all parties and ensure that transferred knowledge is effectively applied. A major challenge that could hinder technology transfer would be the existing issue of follow‐ups after technology transfer initiatives, which were experienced during the COVID‐19 pandemic [146]. For reagent production, technology and knowledge transfer must involve sharing expertise on manufacturing, formulation, analytical methods, and quality control. Achieving this would depend on international collaborations that provide access to advanced knowledge, production protocols, and technologies.

Technology transfer can also help scale up production from research to commercial levels, making high‐quality reagents more affordable for African researchers and healthcare sectors that rely on reagents on a day‐to‐day basis. Key industry players, which mainly involve Asian biomanufacturing companies, can be engaged to set up local manufacturing plants, utilizing raw materials from the region or alternative methods to keep costs low. Previous successes across parts of Africa, where these Asian companies have established pharmaceutical manufacturing facilities, show that these firms can operate effectively in local settings [147]. Also, given their strong capabilities in reagent production, similar investments could be attracted for localized reagent manufacturing. The growth of Africa′s US$25 billion pharmaceutical market [148] further demonstrates that clear demand and supportive policies can draw large‐scale reagent manufacturing, an approach that could likewise reduce import dependence for biomedical reagents. In addition to international partnerships, intra‐African collaboration and knowledge sharing can enhance technology transfer. Learning from biomanufacturing laboratories and institutions in other African countries that have successfully implemented reagent production or biomanufacturing initiatives can help avoid repeated mistakes, speedup implementation, and promote standardized practices across the continent.

### 5.3. Sustainable Funding and Resource Mobilization

A major challenge for biomedical research in Africa is the lack of funding, as most governments do not prioritize financial support for local researchers. This forces scientists to compete for highly competitive international grants. Setting up large‐ and small‐scale reagent production will require strong financial commitment from government‐led partnerships with private investors, international funders, and nonprofit organizations. Private sector partners can also play a role in funding these initiatives. To ensure sustainability, stakeholders must establish clear goals and accountability measures to maximize the impact of funding. In Africa, most research funding comes from international grants and collaborative projects, often focused on disease research, with infectious diseases research leading all grants that come to Africa [149]. Effective funding models must be developed to secure long‐term investment that will provide training for young Africans to serve as valuable human resources that are needed in reagent production facilities. Recent efforts to boost local pharmaceutical manufacturing, such as the Africa CDC′s initiatives and the African Development Bank′s $3 billion commitment over the next decade, have already led to significant growth [150]. With around 600 pharmaceutical companies now operating in Africa, there is a growing demand for locally produced reagents [151]. Many of these companies currently rely on imports, limiting their ability to manufacture beyond drug formulation. Investing in reagent production would not only support pharmaceutical manufacturing but also strengthen research and healthcare systems across the continent.

Biomedical researchers, being at the forefront of scientific advancements in Africa, should take the lead in advocating for local reagent production. They should highlight how this would enhance research capacity, reduce dependence on imports, and position Africa as a leader in biomedical research. The demonstration of these benefits could encourage governments and funding organizations to invest in local manufacturing, ensuring long‐term sustainability and growth in the sector.

## 6. Conclusion

Establishing local reagent production in Africa is critical to strengthening biomedical research, reducing import dependency, and improving healthcare systems. The investment in infrastructure and the provision of robust regulatory frameworks and skilled personnel could allow biomedical researchers on the continent and future biomanufacturing firms to develop cost‐effective and high‐quality reagents tailored to their unique research and healthcare needs. Collaboration between governments, researchers, and private stakeholders will be essential in securing sustainable funding and fostering innovation. With the right support, Africa can lead in biomedical research and diagnostics, ensuring better disease surveillance, treatment, and overall public health outcomes.

## Funding

No funding was received for this manuscript.

## Conflicts of Interest

The authors declare no conflicts of interest.

## Data Availability

Data sharing does not apply to this article as no new data were created or analyzed in this study.
